# 

**Published:** 1855-09

**Authors:** 


PUBLISHED MONTHLY, AT $3 PER ANNUM IN ADVANCE.

### ATLANTA MEDICAL AND SURGICAL JOURNAL.

**JOSEPH P. LOGAN, M. D.,**
Professor of Physiology and General Pathology,
**W. F. WESTMORELAND, M. D.,**
Professor of the Principles and Practice of Surgery.
} EDITORS.

**Pax et scientia, sed veritas sine timore.**

Vol. I. SEPTEMBER, 1855. No. 1.

ATLANTA, GEORGIA:
PRINTED BY C. R. HANLEITER AND CO.
1855.

☞ Each Number contains sixty-four pages. Postage.—Under 500 miles, 15 cents a year; over 500 miles, 30 cents a year—to be pre-paid quarterly. If not pre-paid, double the above rates.

